# Association between Fatigue and Hoehn-Yahr Staging in Parkinson’s Disease: Eight-Year Follow-Up Study

**DOI:** 10.3390/neurolint13020023

**Published:** 2021-05-26

**Authors:** Hiroshi Kataoka, Kazuma Sugie

**Affiliations:** Department of Neurology, Nara Medical University, Kashihara, Nara 634-8522, Japan; ksugie@naramed-u.ac.jp

**Keywords:** Parkinson, fatigue, Hoehn-Yahr stage, risk factor, serotonin, dopamine

## Abstract

The severity of Parkinson’s disease (PD) is developed by multifactorial factors. Falls can worsen disease severity. We previously found that frontal assessment battery (FAB) score was associated with a higher risk of future falls. This eight-year follow-up study aimed to verify whether factors including low FAB score can be the risk of PD progression based on the Hoehn and Yahr scale. In total, 95 patients were initially enrolled in this research and 45 were included in the final follow-up. Then, the cohort was classified into patients with and without disease progression, defined by upgrade of Hoehn-Yahr stage. Differences in clinical characteristics between patients with disease progression and those without were evaluated using the Mann–Whitney U test. Eighteen independent variables were evaluated via a univariate logistic regression analysis. Of the 45 patients enrolled, 32 had disease progression and 13 had no progression. Age (*p* = 0.033), BFI score (*p* = 0.003), Zung self-rating depression (*p* = 0.011), and anxiety scale (*p* = 0.026) were significantly increased in patients who had disease progression than those with no disease progression. On multivariate logistic regression analysis, brief fatigue inventory (BFI) score (OR = 1.048, *p* = 0.045, 95% CI = 1.001–1.098) was significantly related to disease progression. All BFI subscores related to general fatigue. Fatigue could predict the progression of motor dysfunction severity over a longitudinal duration in patients with PD with disease progression, having declining physical and mental fatigue.

## 1. Introduction

Disease severity of Parkinson’s disease (PD) is developed by multifactorial factors. Falls can worsen disease severity. We previously found that slow gait with freezing is associated with a risk for future falls, as assessed using our originally designed, sudden narrowed path [1]. During the two-year course, this cohort had a lower frontal assessment battery (FAB) score, which was closely correlated with slow gait with freezing, and it could be a predictor of a higher risk of falling in 30 patients with Hoehn and Yahr stage III PD [2]. To determine the risk of future falls, we have previously investigated 100 patients with PD, including this cohort. Results showed that the low FAB score and a history of falls within the last six months were associated with a higher risk of future falls [3]. This finding can give rise to an open question of whether factors including low FAB score can be predictors of PD severity during a longitudinal follow-up period. We assessed 100 patients during an eight-year follow-up to determine the predictors of PD progression.

## 2. Methods

Methods used in the present study were the same as our previous study [3]. This study included 100 consecutive patients who fulfilled the UK PD Society Brain Bank criteria [4]. In addition, no patient had the following conditions: possible or probable multiple system atrophy per the Gilman criteria [5] and progressive supranuclear palsy per the National Institute of Neurologic Disorders and Stroke-SPSP diagnostic criteria [6]. No patient had any other form of parkinsonism, such as vascular parkinsonism, large vessel disease, infarction, or tumors on magnetic resonance imaging and evidence of psychosis, epilepsy, a history of stroke, transient ischemic attacks, uncompensated heart failure, severe pulmonary problems, or a history of surgical intervention like deep brain stimulation surgery. All patients could follow our instructions. All patients were evaluated upon enrollment using the following scales: scores on parts I, II, III, and IV of the unified Parkinson’s disease rating scale (UPDRS) [7], Hoehn-Yahr stage [8], mini-mental status examination (MMSE), FAB, brief fatigue inventory (BFI) [9], Zung self-rating depression and anxiety scales, Parkinson’s disease sleep scale (PDSS), and Tinetti balance and gait assessment [10]. The history of falls during the preceding 6 months was ascertained at enrollment. The daily dose of antiparkinsonian medications was converted into the equivalent dose of levodopa per previous studies [3,11,12]. Disease progression was defined by upgrade of Hoehn-Yahr stage for 8 years.

**Figure 1:**
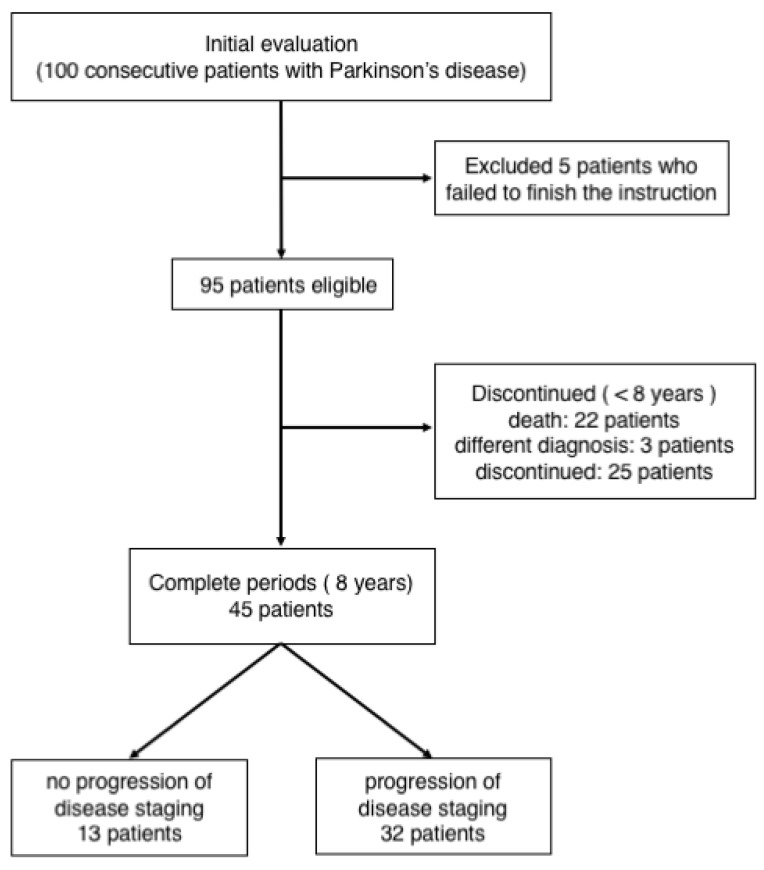
Patient selection procedure.

All the 95 patients with PD (Figure 1) registered for the study. These patients visited our hospital every 1–3 months during the 8-year follow-up. During the follow-up, 22 patients died, and 25 patients who required hospitalization and were transferred to other hospitals or did not return to our hospital were excluded from the study. In addition, three patients were eventually diagnosed as having other diseases (normal pressure hydrocephalus in one and progressive supranuclear palsy–parkinsonism in two). The remaining 45 patients were included in data analysis. The study protocol was approved by the Medical Ethics Committee of Nara Medical University.

### Statistical Analysis

The statistical significance of differences in clinical characteristics between patients with and without disease progression defined by a change of Hoehn-Yahr stage was evaluated using the Mann–Whitney test. The disease progression was coded as objective variable (present = 1). Overall, the following 18 independent variables were evaluated using univariate logistic regression analysis: age, gender (men = 1), disease duration, levodopa equivalent dose, UPDRS total score, UPDRS part I, UPDRS part II, UPDRS part III, UPDRS part IV, Tinetti balance, Tinetti gait, MMSE, FAB, BFI, Zung self-rating depression scale, Zung self-rating anxiety scale, summed score of items on PDSS that were rated from good to bad sleep, and history of falls (absent = 0, present = 1). Variables that were significantly related to disease progression (*p* < 0.05) were entered into multivariate logistic regression analysis using forced entry. Odds ratios (OR) and 95% confidence intervals (CI) were calculated. In addition, the correlations of each variable were evaluated using Spearman’s rank correlation test. The SPSS software (version 18) was used for statistical analysis.

## 3. Results

Of the 45 patients enrolled, 32 had disease progression and 13 had no progression (Table 1). Age (*p* = 0.033), BFI score (*p* = 0.003), Zung self-rating depression (*p* = 0.011), and anxiety scale (*p* = 0.026) were significantly increased in patients who had disease progression than those with no disease progression (Table 2). On univariate analysis, BFI score (OR = 1.057, *p* = 0.008, 95% CI = 1.015–1.102), Zung self-rating depression score (OR = 1.148, *p* = 0.017, 95% CI = 1.025–1.285), and anxiety scale (OR = 1.143, *p* = 0.036, 95% CI = 1.009–1.295) were significantly related to disease progression (Table 3). The results of multivariate logistic regression analysis showed that fatigue (as measured by BFI score) significantly increased the risk of disease progression (OR = 1.048, *p* = 0.045, 95% CI = 1.001–1.098). All BFI subscores related to general fatigue (*p* < 0.001), mood (*p* = 0.014), walking ability (*p* = 0.014), normal work (*p* = 0.002), interpersonal relationships (*p* = 0.015), and enjoyment of life (*p* = 0.013) were significantly higher in patients with disease progression (Table 4). The baseline characteristics between patients who were included in and those who dropped out from the study are shown in Table 5.

## 4. Discussion

The present study showed that patients with disease progression measured by Hoehn-Yahr stage have higher fatigue scores than those without progression. Therefore, fatigue was a significant predictor for disease progression.

Fatigue has an estimated prevalence of 50% in patients with PD [13] and is an intrinsic symptom derived from the pathobiological mechanism of the disease [13,14], being presence even before the onset of motor symptoms [15,16,17]. Fatigue tends to persist over the disease course and worsens with disease progression [18]. Notably, in PD, fatigue was noted to be related to striatal dopamine deficiency and an imbalance between both dopaminergic and serotonergic neurotransmitters [14]. Typically, in PD, the serotonergic function reduces in the basal ganglia and limbic structures, and this finding is possibly associated with fatigue [14]. A pathological process of PD might probably arise from the olfactory bulb and spread toward the limbic areas, potentially causing fatigue [13]. In addition, another process might arise from the dorsal vagal nucleus and ascend to the substantia nigra, causing the degeneration of the nigrostriatal dopaminergic system [19]. These pathological processes can increase the severity of both motor symptoms and fatigue. Most studies have yielded conflicting results regarding the correlation between fatigue and PD progression [13,14]. Most studies revealed that fatigue did not correlate with motor disability, but 13 cross-sectional studies demonstrated that fatigue was associated with a higher Hoehn-Yahr stage [13]. A meta-analysis revealed that patients with fatigue had  Hoehn-Yahr state 0.33 points higher than those without fatigue [13]. Moreover, compared to PD patients without fatigue, patients with PD and fatigue exhibited more muscle fatigability during a finger-tapping task [20] and low-level leisure physical activity [21]. Therefore, a correlation seems to exist between fatigue and PD progression.

Notably, there are limited number of longitudinal studies that investigated the association between motor progression and fatigue. One study compared the non-motor symptoms and motor severity at baseline and two years later and observed no significant differences in fatigue levels obtained using a semi-structured 12-domain interview between patients with stable versus worsened Hoehn-Yahr stage [22]. However, the PD cohort of this study was similar to that of the PRIMO study, with the cross-sectional PRIMO study revealing fatigue severity increased with the Hoehn-Yahr stage progression [18]. Another study with a design similar to ours evaluated motor severity and non-motor symptoms of patients with PD at baseline, and after two years to determine a predictor for the motor progression based on Movement Disorders Society (MDS)-UPDRS scale [23]. Fatigue, assessed by the Fatigue Severity Scale, was found to increase the risk for MDS-UPDRS part 3 score progression, thereby indicating that subjective fatigue severity score could predict motor function in PD. This study was mentioned as the first study to have focused on the association between fatigue and motor function. Another prospective study tried to determine demographic and clinical properties associated with change in fatigue severity scale score, but could not detect any motor dysfunction progression based on UPDRS score or Hoehn-Yahr stage [24]. The reason for this finding could be that the follow-up period of one year was too short, and patients with fatigue were given higher doses of dopaminergic medications during follow-up. The dopaminergic medication doses are typically increased with motor dysfunction progression; therefore, the finding might indirectly suggest an association between fatigue and Hoehn-Yahr stage.

The pathogenesis of fatigue is unknown. Serotonin is closely associated with fatigue in chronic fatigue syndrome [25,26]. In patients with PD, the postmortem brain tissue has pathologically revealed degeneration of both dopaminergic and serotonergic neurons [27,28]. PET studies using markers of dopamine storage capacity and serotonin transporter in PD patients with fatigue have revealed serotonergic denervation in the basal ganglia and the limbic structures compared with non-fatigued patients [29]. Furthermore, serotonergic neurons, instead of the dopaminergic neurons, are known to produce dopamine with the progression of PD, especially in patients with dyskinesia. Therefore, dopaminergic and serotonergic neurons are closely related to PD. The spread of a neuropathological process toward basal ganglia and limbic structures closely associated with dopaminergic and serotonergic neurons could contribute to dysfunction of prefrontal-basal ganglia loops causing fatigue [30,31]. A higher degree of subjective fatigue severity could reflect the widespread presence of α-synuclein, which influences the progression of motor dysfunction.

The critical distinction between the subjective perception of fatigue and performance fatigability can help understand the fatigue of neurological illness [32]. The former involves homeostatic and psychological factors, with hypothalamic lesion and frontal dysfunction in PD [14,33]. On the other hand, the latter involves peripheral and central factors. Measuring quadriceps performance in PD revealed a strong negative correlation between central activation deficits and quadriceps strength, thereby suggesting the absence of muscle fatigue [34]. Nevertheless, central fatigue involves both physical and mental components, and several physiological mechanisms were hypothesized to cause dysfunction of frontal striothalamocortical loops and imbalance among various neurotransmitters [14]. The present study documented decreased scores on all BFI subscales, including physical and mental fatigue components, which might indicate that fatigue is influenced by interactions among these factors, specifically central fatigue is considered to be highly influenced by psychological factors [14].

The present study demonstrates an association between fatigue and motor dysfunction severity as previously documented in patients with a longer follow up than previous studies [18,23]. We recognize some limitations of the study. Indeed, a specific Parkinson’s fatigue scale was not used. Also, knowledge about the relationship between fatigue and depression can be better evaluated using the quick inventory of depressive symptomatology [35] or the Hamilton depression scales [36] compared with the Zung self-rating depression score, which was used in the current study. Another limitation was the small number of patients who did not demonstrate progression in motor staging.

In conclusion, fatigue could predict the progression of motor dysfunction severity over a longitudinal duration in PD patients with disease progression, having declining physical and mental fatigue.

## Figures and Tables

**Table 1:** Hoehn-Yahr stage of subjects with Parkinson’s disease in our study.

Patients with Parkinson’s Disease	Hoehn-Yahr Stage at the Entry	Hoehn-Yahr Stage after 8 Years
Total (*n* = 45) (mean, SD, range)	2.8, 0.6, 1–4	4.0, 0.9, 2–5
no disease progression ^a^ (*n* = 13)	2.9, 0.6, 2–4	2.9, 0.6, 2–4
disease progression ^a^ (*n* = 32)	2.8, 0.6, 1–4	4.5, 0.5, 4–5

^a^ disease progression defined by an upgrade of Hoehn-Yahr stage.

**Table 2:** Basic characteristics of subjects with Parkinson’s disease in our study.

During 8 Years	Total (*n* = 45)	No Disease Progression ^a^ (*n* = 13)	Disease Progression ^a^ (*n* = 32)	*p*
Age (years), mean, SD	69.0, 8.1	65.8, 7.0	70.3, 8.2	0.033 *
men, *n*, %	17, 37.7	4, 30.7	13, 40.6	0.737
Disease duration (months), median, range	40, 2–329	61, 6–101	38, 2–329	0.831
Levodopa equivalent dose (mg/day), median, range	207.4, 0–998	202.2, 0–998	206.6, 0–600	0.85
UPDRS total score, mean	40.6, 6.8	41.0, 17.1	40.4, 16.9	0.783
UPDRS part 1, median, range	1.7, 0–13	1.4, 0–13	1.8, 0–8	0.46
UPDRS part 2, mean	11.7, 6.6	10.9, 6.2	12.0, 6.8	0.716
UPDRS part 3, mean	25.2, 9.7	27.1, 10.7	24.5, 9.3	0.415
UPDRS part 4, median, range	0.69, 0–9	0.4, 0–9	0.8 (0–9)	0.17
Tinetti balance, mean	9.8, 3.2	10.6, 3.3	9.5, 3.1	0.089
Tinetti gait, mean	12.5, 3.3	13.4, 3.4	12.1, 3.5	0.194
Mini-Mental Status Examination, mean	27.5, 2.6	27.1, 3.6	27.7, 2.1	0.889
Frontal Assessment Battery, mean	14.2, 2.9	14.2, 3.8	14.3, 2.5	0.613
Brief fatigue inventory, mean	29.0, 20.9	15.0, 13.5	34.6, 20.8	0.003 *
Zung Self-Rating Depression scale score, mean	43.7, 7.3	39.4, 5.9	45.5, 7.1	0.011 *
Zung Self-Rating anxiety scale score, mean	37.9, 6.2	34.6, 7.9	39.2, 5.0	0.026 *
Parkinson’s disease Sleep Scale, mean	1047.6, 287.7	1111.4, 231.9	1021.7, 307.1	0.46
History of fall present ^b^	15, 33.3	4, 30.7	11, 34.3	0.553

H-Y: Hoehn-Yahr stage, UPDRS: Unified Parkinson’s Disease Rating Scale, PD: Parkinson’s disease. ^a^ disease progression defined by an upgrade of Hoehn-Yahr stage, ^b^ during 6 months before entry, * *p* < 0.05.

**Table 3:** Independent predictive variables for disease progression defined by an upgrade of Hoehn-Yahr stage.

	Crude Odds Ratio (95% CI)	*p*	Adjusted Odds Ratio (95% CI) ^a^	*p*
Age (years)	1.072 (0.983 to 1.168)	0.114		
gender	1.539 (0.390 to 6.076)	0.538		
Disease duration (months)	1.003 (0.990 to 1.017)	0.637		
Levodopa equivalent dose (mg/day)	0.998 (0.995 to 1.002)	0.381		
UPDRS total score	0.998 (0.960 to 1.037)	0.902		
UPDRS part 1	1.024 (0.798 to 1.314)	0.854		
UPDRS part 2	1.027 (0.927 to 1.139)	0.61		
UPDRS part 3	0.972 (0.908 to 1.040)	0.406		
UPDRS part 4	1.055 (0.817 to 1.361)	0.682		
Tinetti balance	0.869 (0.673 to 1.122)	0.281		
Tinetti gait	0.883 (0.704 to 1.106)	0.278		
Mini-Mental Status Examination	1.085 (0.852 to 1.381)	0.509		
Frontal Assessment Battery	1.010 (0.810 to 1.259)	0.932		
Brief fatigue inventory	1.057 (1.015 to 1.102)	0.008 *	1.048 (1.001 to 1.098)	0.045 *
Zung depression score	1.148 (1.025 to 1.285)	0.017 *	1.102 (0.944 to 1.286)	0.218
Zung anxiety score	1.143 (1.009 to 1.295)	0.036 *	1.011 (0.852 to 1.200)	0.898
Parkinson’s disease Sleep Scale (PDSS)	0.999 (0.996 to 1.001)	0.344		
History of fall present ^b^	1.179 (0.295 to 4.710)	0.816		

UPDRS: Unified Parkinson’s Disease Rating Scale, PD: Parkinson’s disease. ^a^ adjusted for Frontal Assessment Battery, Zung depression score, and Zung anxiety score, ^b^ during 6 months before entry, * *p* < 0.01.

**Table 4:** Comparative subscores of Brief Fatigue Inventory of patients with PD with and without disease progression defined by an upgrade of Hoehn-Yahr stage.

	No Progression of H-Y Staging (*n* = 13)	Progression of H-Y Staging (*n* = 32)	*p*
General fatigue (mean, median, range)	0.75, 0–3	4.5, 0–10	<0.001 *
Mood	1.0, 0–5	4.09, 0–10	0.014 *
Walking ability	1.0, 0–5	4.09, 0–10	0.014 *
Normal work *	1.57, 0–4	5.2, 0–10	0.002 *
Relations with other people	0.75, 0–3	2.85, 0–8	0.015 *
Enjoyment of life	0.75, 0–3	3.75, 4, 0–10	0.013 *

H-Y: Hoehn-Yahr stage, *: *p* < 0.05.

**Table 5:** Baseline characteristics between patients with Parkinson’s disease who were included in and those who dropped out from the study.

During 8 Years	Included Patients (*n* = 45)	Dropped Patients (*n* = 50)	*p*
Age (years), mean, SD	69.0, 8.1	74.4, 7.1	0.001 *
men, *n*, %	17, 37.7	27, 54	0.084
Disease duration (months), median, range	40, 2–329	30, 4–244	0.558
Levodopa equivalent dose (mg/day), median, range	207.4, 0–998	232.7, 0–585	0.436
UPDRS total score, mean	40.6, 16.8	39.4, 21.4	0.712
UPDRS part 1, median, range	1.7, 0–13	2.3, 0–9	0.341
UPDRS part 2, median, range	10, 3–30	10, 0–32	0.476
UPDRS part 3, mean	25.7, 9.7	24.5, 13.8	0.519
UPDRS part 4, median, range	0.69, 0–9	0.62, 0–8	0.573
Tinetti balance, median, range	11.2, 0–12	10.8, 1–16	0.54
Tinetti gait, median, rang	13.8, 3–16	13.2, 0–18	0.405
Mini-Mental Status Examination, mean	27.5, 2.6	25.8, 3.6	0.016 *
Frontal Assessment Battery, mean	14.2, 2.9	12.8, 3.4	0.039 *
Brief fatigue inventory, mean	29.0, 20.9	36.9, 22.3	0.085
Zung Self-Rating Depression scale score, mean	43.7, 7.3	44.7, 7.3	0.437
Zung Self-Rating anxiety scale score, mean	37.9, 6.2	38.6, 7.6	0.762
Parkinson’s disease Sleep Scale, median, range	1085, 73–1446	1103, 92–1446	0.905
History of fall present ^a^	15, 33.3	24, 48	0.107

H-Y: Hoehn-Yahr stage, UPDRS: Unified Parkinson’s Disease Rating Scale, PD: Parkinson’s disease, ^a^ during 6 months before entry, * *p* < 0.05.

## Data Availability

The data that support the findings of this study are openly available in this article and available from the corresponding author upon reasonable.
